# State-dependent mortality can enhance behavioral unpredictability

**DOI:** 10.1186/s12898-020-00303-9

**Published:** 2020-06-25

**Authors:** Toshinori Okuyama

**Affiliations:** Department of Entomology, National Taiwan University, No. 1, Sec. 4, Roosevelt Rd., Taipei, 106 Taiwan

**Keywords:** Within-individual variation, Foraging, Stochastic dynamic programming, Risk-sensitivity

## Abstract

**Background:**

Although behavioral unpredictability is widely described within-individual variability in behavior, its adaptive significance is little understood. Using a dynamic state variable model, this study investigated the conditions under which behavioral unpredictability (a component of within-individual variability) in foraging behavior is advantageous. The model considers a situation in which a forager forages for a fixed period, represented by discrete time steps. The outcome of foraging may change the level of a state (e.g., size and fat storage) of the forager at each time step, and variability in the foraging outcome is assumed to be positively correlated with behavioral unpredictability. The probability of death at each time step is influenced by the state at the same time step. Reproduction occurs after all the foraging steps and is influenced by the state level of a forager at the time of reproduction. According to the expected utility hypothesis, the relationship (e.g., curvature) between the state and fitness will determine the role of behavioral unpredictability. In the model, the relationship was obtained by using the backward iteration method for each foraging time step.

**Results:**

State-dependent mortality adds curvature to the relationship between the state and fitness, which makes the effect of behavioral unpredictability on fitness either positive or negative. This conclusion holds for any state-dependent mortality (i.e., as long as mortality is not independent of the state factor). Given that state-dependent mortality is commonly described, conditions that benefit behavioral unpredictability are likely also common.

**Conclusions:**

When mortality depends on a state that is influenced by behavior, conditions that favor behavioral unpredictability may become common. How behavioral unpredictability influences the variability of behavioral outcomes is as important as how it influences the expectation of behavioral outcomes when studying the adaptive significance of behavioral unpredictability.

## Background

When an animal is repeatedly observed, its behavioral expressions can be highly variable. This within-individual variability in behavior can be categorized into phenotypic plasticity and behavioral unpredictability [1–4]. Phenotypic plasticity explains within-individual variability observed along some environmental gradients such as wind velocity, predation risk and temperature [5]. Behavioral unpredictability (also known as “residual behavioral variance”) is the remaining (residual) variability after the effects of environmental and explanatory variables are accounted for [1–4]. Although behavioral unpredictability may appear as mere noise in behavioral expressions, there are some patterns in expressions of behavioral unpredictability. For example, in the stickleback, *Gasterosteus aculeatus*, shy individuals are more unpredictable than bold individuals [6]. Behavioral unpredictability of nestling caring birds decreases with the age of nestling [2]. Furthermore, behavioral unpredictability can be a heritable trait [7], suggesting that observed patterns in behavioral unpredictability may be the result of natural selection. Notwithstanding, the adaptive significance of behavioral unpredictability is little understood. A commonly used example of the function of behavioral unpredictability is the protean behavior of prey. In the ecological literature, the term protean is commonly interpreted as unsystematic [8] or unpredictable [9], and protean prey exhibit erratic escape trajectories (e.g., changing escape direction abruptly) upon encountering predators, which makes it difficult for predators targeting them [9–11]. However, protean escape behavior may be a restrictive example in which unpredictability is quantified in a single predator encounter (e.g., variation in the turning angle in an escape trajectory) as opposed to variability in escape strategies in multiple predator encounters. Explanations for adaptive significance of behavioral unpredictability are needed for a wider ecological scenarios.

**Fig. 1:**
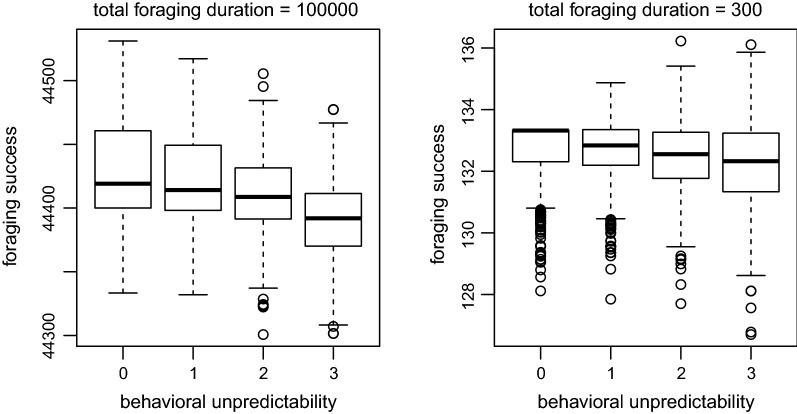
Relationship between foraging success and behavioral unpredictability in patch residence time. Each boxplot summarizes simulation results from 1000 foragers. A forager’s patch search times (i.e., durations needed to find a patch) were generated from a gamma distribution with mean $\lambda ^{-1}$ and variance $\sigma _{s}^2$, and patch residence times were generated from a gamma distribution with mean $t^*=\sqrt{\lambda b}/\lambda$ and standard deviation $\sigma _{t}$ that represents behavioral unpredictability. When foraging success is evaluated in a short duration (300 time units, right figure), behavioral unpredictability increases variability in behavioral outcome while having a minor effect on the expected behavioral outcome. This pattern disappears when foraging success is evaluated in a long duration (100,000 time units, left figure). $\lambda =0.01, a=100, b=25, \sigma _{s}^2=5$

One possible consequence of behavioral unpredictability is increased variability in the outcome of the behavior. To illustrate this point, a simple simulation model is used here. The model considers the marginal value theorem [12], in which foragers forage in a patchy environment. For simplicity, all patches are assumed to be equal, and the patch encounter rate is $\lambda$. A forager’s energy gain *g* in a patch increases as its patch residence time *t* increases, which is described by $g(t)=at/(b+t)$ where *a* and *b* are parameters that influence the relationship. Then, the optimal patch residence time can be shown as $t^*=\sqrt{\lambda b}/\lambda$. When an individual’s patch residence times are variable for each patch and follow a probability distribution with mean $t^*$ and standard deviation $\sigma _{t}$, $\sigma _{t}$ can be interpreted as behavioral unpredictability such that foragers with $\sigma _{t}=0$ will always spend an exact duration of $t^*$ in each patch. Simulation results show that when the total foraging duration is short, behavioral unpredictability can increase the variation in foraging success (Fig. 1) (the R script is available in Additional file 1). This pattern can emerge as a combination of environmental stochastically and a finite duration in which the performance is evaluated. Environmental stochastically creates variable experiences among individuals (e.g., some foragers find more patches than others by chance), and this variability may be pronounced in a short duration, which may be intuitively understood using a coin-flip analogy. For example, if we periodically flip a fair coin, flipping the coin 4 times (i.e., representing a short duration) and getting 0 or 4 heads is not unexpected, but if we flipped it 40 times (i.e., representing a long duration) getting 0 or 40 heads would be practically impossible. In the patch foraging example, the optimal patch residence time is different for each individual’s experience. For example, a forager that finds only one patch should stay in the patch for a long duration to fully utilize the patch, while a forager that finds many patches should spend a shorter duration in each patch. Behavioral unpredictability can produce both suitable and unsuitable behavioral responses by chance, which increases variability in the behavioral outcome. When the foraging success is evaluated in a long duration, such a pattern can disappear (Fig. 1).

When behavioral unpredictability is associated with variability in behavioral outcome, behavioral unpredictability may be considered as risk-prone behavior, with risk representing uncertainty [13]. The sequence from behavior to fitness can be shown as

$$\begin{aligned} \text {behavior}~X \rightarrow \text {behavioral outcome}~Y \rightarrow \text {fitness}~W \end{aligned}$$

where *X*, *Y*, and *W* represent the respective random variables. In the above patch foraging example, behavior *X* is the patch residence time, and behavioral outcome *Y* is the total amount of energy gained. As shown in the simulation example, Var(*X*) (i.e., behavioral unpredictability) and Var(*Y*) may be positively correlated (Fig. 1). The relationship between behavioral outcomes and fitness is described by

1 $$\begin{aligned} w = \Phi (y) \end{aligned}$$

where the lowercase letters (*w* and *y*) represent realizations of the respective random variables *W* and *Y*. On the basis of the expected utility hypothesis [13], which is largely equivalent to Jensen’s inequality, the effect of variability in the behavioral outcome Var(*Y*) on the expected fitness *E*(*W*) depends on the shape of the fitness function (Eq. 1). In particular, when $\Phi (y)$ is convex, Var(*Y*) has a positive influence on *E*(*W*), which may also select variability in behavior Var(*X*) when Var(*Y*) and Var(*X*) are positively correlated. When $\Phi (y)$ is concave, Var(*Y*) has a negative effect on *E*(*W*). When $\Phi (y)$ is linear, Var(*Y*) has no influence on *E*(*W*). As such, understanding the curvature of $\Phi (y)$ may reveal the effect of Var(*Y*) on fitness, which can link to behavioral unpredictability when Var(*X*) and Var(*Y*) are correlated.

In the case of foraging, Eq. 1 represents how foraging success (*y*) relates to reproduction (*w*). Theoretical population models typically assume that foraging success (i.e., functional response) and reproduction are linearly related (e.g., the Lotka-Volterra model and its many variants). Empirical numerical response studies often show that the relationship between prey density and reproduction is concave [14–16], but it is difficult to relate these data to Eq. 1 because prey density does not equal foraging success. When body size is considered as a surrogate of behavioral outcome (e.g., successful foraging outcomes lead to larger body sizes), the relationship between body size and fecundity may be linear or convex [17–20]. As such, Eq. 1 is likely system-specific and may take any form.

The relationship (Eq. 1) at the time of reproduction is not the only issue when applying the expected utility hypothesis to understand the effect of behavioral unpredictability on fitness. When reproduction is used as the surrogate of fitness *w* in Eq. 1, the corresponding *y* is the state at the time of reproduction. However, the same relationship may not hold at different time points (e.g., *y* for immature stages). For example, even when large individuals (e.g., *y* representing size) are expected to have higher fitness than small individuals at the time of reproduction, small individuals may have higher expected fitness than large individuals when they are juveniles (e.g., large juveniles have low expectations of survival to reproduction due to size-dependent predation). Although such state-dependent mortality has been routinely incorporated in previous models to study time and state-dependent optimal strategies [21–26], its effect on the curvature of the fitness function has not been considered in light of behavioral unpredictability. This study examined the effect of behavioral unpredictability on fitness by focusing on the time-dependent relationship between the state and expected fitness (i.e., equivalent of Eq. 1 when *y* represents the state at various life stages) using a method of dynamic state variable models [23, 24].

## Results

### Isolated effects of state-dependent mortality

**Fig. 2:**
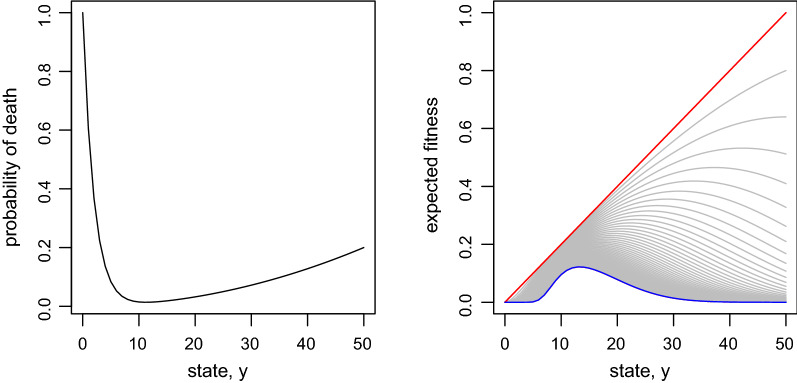
State-dependent mortality (left) and its effect on the expected fitness (right). The red line is the terminal fitness $\Phi _{0}(y)$. The blue line is $\Phi _{\tau }(y)$ when $\tau =50$. Gray lines are $\Phi _{\tau }(y)$ when $\tau$ is between 1 and 49. $\Phi _{\tau }(y)$ decreases monotonically with $\tau$ such that the $\tau$th gray line below the red line is $\Phi _{\tau }(y)$

**Fig. 3:**
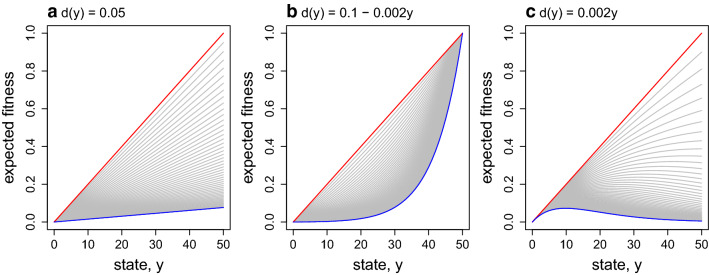
Effects of state-dependent mortality on expected fitness. **a** State-independent: $d(y)=0.05$. **b** State-dependent: $d(y)=0.1-0.002y$. **c** State-dependent: $d(y)=0.002y$. The red line is the terminal fitness $\Phi _{0}(y)$. The blue line is $\Phi _{\tau }(y)$ when $\tau =50$. Gray lines are $\Phi _{\tau }(y)$ when $\tau$ is between 1 and 49. $\Phi _{\tau }(y)$ decreases monotonically with $\tau$ such that the $\tau$th gray line below the red line is $\Phi _{\tau }(y)$

In the absence of behavioral unpredictability and state level dynamics (i.e., $q=0$), nonlinearity in the expected fitness $\Phi _{\tau }(y)$ will result regardless of the state-dependent mortality *d*(*y*) is nonlinear (Fig. 2) or linear (Fig. 3). Under this simple assumption, $\Phi _{\tau }(y)$ can be explicitly derived without the backward iteration: $\Phi _{\tau }(y)=s_{\tau }(y)\Phi _{0}(y)$ where $s_{\tau }(y)=(1-d(y))^{\tau }$ is the probability that a forger of state *y* will survive $\tau$ time steps (i.e., until reproduction). From the expression, it is clear that nonlinearity in $\Phi _{\tau }(y)$ emerges because $s_{\tau }(y)$ is nonlinear simply due to the multiplicative nature of survival processes.

As $\tau$ increases, the shape of the expected fitness function changes from linear to unimodal curve under Eq. 2 (Fig. 2). The unimodal curve is locally concave around the mode (promote predictability), and both sides are convex (promote unpredictability) if *q* were a flexible trait. This pattern is qualitatively similar to the linear mortality function where the mortality increase with state level (Fig. 3c). Under these scenarios, individuals with high state levels are unlikely to survive when the time till reproduction $\tau$ is long. This negative effect of *y* on fitness (through survival) and the positive effect of *y* on fitness (through reproduction) creates the unimodal shape in the expected fitness.

Although the specific mortality functions were examined here, due to this simple mechanism, nonlinearity in $\Phi _{\tau }(y)$ will result as long as mortality depends on state (an example where mortality is independent of the state is shown in Fig 3a). The same can be said about the parameter values. For example, the maximum state-dependent mortality was set as 0.2. This value depends on the actual time unit of discrete time step. When foraging durations are subdivided in shorter time units, then mortality in each time unit will decrease, and vice versa. However, altering these values will only change the rate of change (i.e., lower mortality will require greater $\tau$ to see equivalent results), and qualitative pattern will not change.

### Full model

**Fig. 4:**
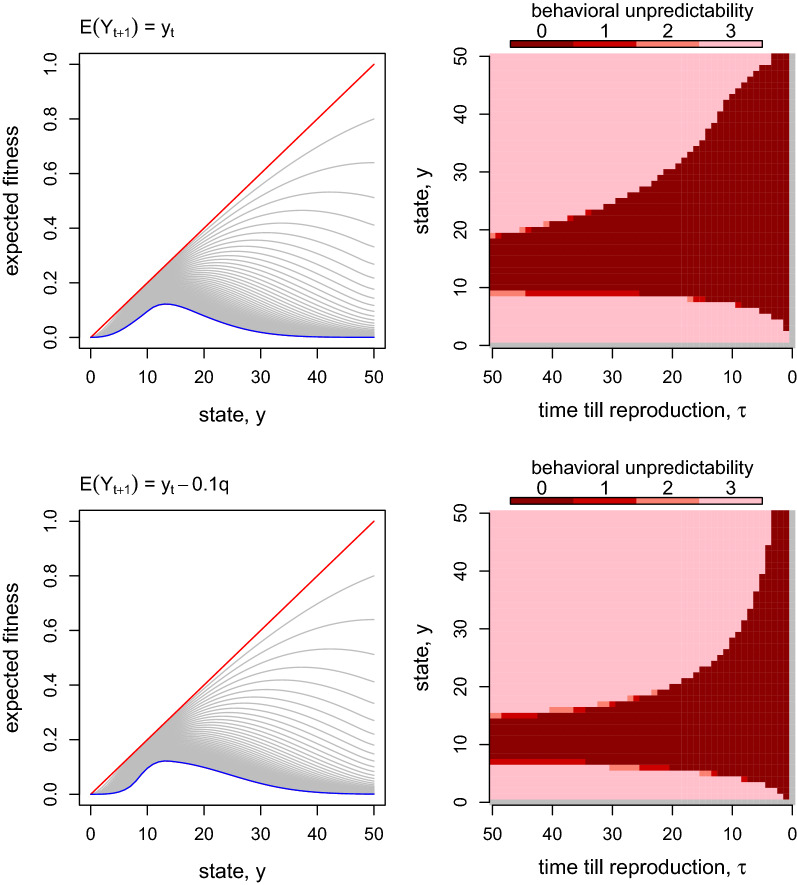
Time-dependent expected fitness (left column) and optimal behavioral unpredictability (right column). Top figures correspond with a case where behavioral unpredictability has no effect on the expected behavioral outcome ($u=0$). The bottom figures correspond with a case where behavioral unpredictability *q* has a negative effect on the expected behavioral outcome ($u=-0.1$). The interpretation of the left column figures is the same as that for Figs. 2 and 3. In the right column figures, grey color is used for $\tau =0$ and $y=0$ because $\tau =0$ is at reproduction and foragers no longer forage, and $y=0$ represents deterministic death (Eq. 2)

Inclusion of behavioral unpredictability ($q\in \{0,1,2,3\}$) as well as the optimal foraging consideration have little effect on the shape of $\Phi _{\tau }(y)$ (Fig. 4). The model was analyzed for two cases. The first case is where behavioral unpredictability does not influence expected foraging gain ($u=0$, Fig. 4 top). The second case is where behavioral unpredictability decreases expected foraging gain while increasing variability in foraging gain ($u=-0.1$, Fig. 4 bottom). In both cases, the dynamics of the shape of fitness function is similar to the simplified example (comparison of Fig. 2 and Fig. 4 left column). High levels of unpredictability is selected for high and low state levels, and 0 unpredictability (i.e., complete predictability) is selected at intermediate state levels. As discussed above, this results come from the unimodal shape of the expected fitness function.

The negative effect behavioral unpredictability on the expected foraging gain (i.e., $u=-0.1$) has both positive and negative effects on the selection on behavioral unpredictability. The right tail of the unimodal fitness function (corresponding with high state levels when $\tau$ is sufficiently large) is convex and thus variability in foraging success is selected. In the same region, the state has a negative effect on expected fitness (i.e., $\Phi _{\tau }(y)$ has a negative slope in *y*). When behavioral unpredictability has no effect on expected foraging success ($u=0$), it has no effect on expected fitness thorough the expected foraging gain. On the other hand, when behavioral unpredictability has a negative effect on the expected foraging success, it has a positive effect on expected fitness through the expected foraging gain. Consequently, the region where behavioral unpredictability is selected expands when $u=-0.1$ compared to $u=0$ for high state levels (Fig. 4).

The left tail of the unimodal fitness curve is also convex, and the state level has a positive effect on expected fitness (i.e., $\Phi _{\tau }(y)$ has a positive slope in *y*) in low state levels. Consequently, when behavioral unpredictability has a negative effect on expected foraging success ($u=-0.1$), it also has a negative effect on expected fitness. As results, the region where behavioral unpredictability is selected shrinks when $u=-0.1$ compared to $u=0$ for low state levels (although the difference is small in Fig. 4). In other words, when behavioral unpredictability influences both the expectation and variability of behavioural outcome, the net effect of behavioural unpredictability on fitness is determined by the combination of the effects through the expectation and variability.

## Discussion

When behavioral unpredictability is related to the variability of behavioral outcomes (e.g., Fig. 1), its effect on fitness depends on the relationship between behavioral outcomes and fitness (i.e., the curvature of $\Phi _{\tau }(y)$ in the model). This study demonstrated that the curvature of $\Phi _{\tau }(y)$ is influenced by state-dependent mortality *d*(*y*) through multiplicative survival processes. The mechanism is simple and generally relevant to any *d*(*y*) as long as *d*(*y*) is not independent of state *y*. Classical risk-sensitivity studies explain why animals are prone to engaging in risky behavior when their energy state is low based on the expected utility hypothesis (e.g., Eq. 1 tends to be convex when organisms are under poor conditions) [13]. However, an important result of this study is that such selection for risky (or unpredictable) behavior is not restricted to organisms under poor conditions.

State factors *y* that are relevant to this study are those influenced by foraging success while influencing mortality. One possible state is starvation/satiation. However, its influence on mortality may be system-specific. For example, starvation may influence essential physiological processes, such as thermoregulation and metabolism [27], which may relate to mortality. On the other hand, e.g., satiation impairs locomotive ability in mosquitoes and increases the risk of predation [28]. Fat storage used in the study is another potential state with associated benefits (e.g., energy storage, body insulation, and social signals) and costs (e.g., fat-dependent metabolism and predation risk) [29]. Size is yet another potential variable, with numerous examples of size-dependent predation where predators preferentially attack prey in a specific size range [30–32]. If we consider size as the state, it may be unlikely that size (not considering weight) decreases with unsuccessful foraging as assumed in the model, but this is not a problem because the result (i.e., emergence of nonlinearity) does not depend on the assumption that unsuccessful foraging reduces the state level (e.g., Fig. 3). Furthermore, empirically documented terminal fitness functions $\Phi _0(y)$ are often convex when size is considered [17–19], which renders behavioral variability more advantageous in general when the state level *y* relates to size.

While there may be a number of state variables that are influenced by foraging and influence mortality, how fast a variable changes due to foraging success is also an important detail. This study assumed a positive correlation between behavioral unpredictability and variability in behavioral outcomes. It also assumed that the potential negative effect of behavioral unpredictability on expected behavioral outcome is not so strong such that most successful (and most unsuccessful) individuals come from ones with high unpredictability (Fig. 1). Although these relationships come from the simple mechanism discussed above, and the relationship can be established in various models such as the one used in Fig. 1, the relationship will not always hold. Even in the example used in Fig. 1, it depends on the foraging duration considered. When state variable *y* rapidly changes foraging success, the assumptions are more likely satisfied. For example, previous models concerning fat reserve of small birds used one day as the unit of fat reserve dynamics [22, 24], which may be a short enough time period in which environmental stochastically can create sufficient variability (that interacts with behavioral unpredictability) among individuals. Similarly, the mass of female mosquitoes can significantly increase after one feeding bout [28]. On the other hand, body size (discussed above) is expected to be a much slower variable than body mass (e.g., organisms that grow through moulting). Empirical studies that examine the relationship between behavioral unpredictability and the mean and variance of behavioral outcomes with various state variables will be useful. When the assumed correlation is empirically established, the approach used here can be used to build a system specific model to study observed patterns in behavioral unpredictability.

## Conclusions

When considering the function of a trait including behavioral unpredictability, it is conventional to consider how the trait improves upon the expectation of behavioral success. Instead, this study focused on the relationship between behavioral unpredictability and the variability (rather than expectation) of behavioral success and showed that even when a strategy with greater behavioral unpredictability does not positively influence average foraging success, it may still be advantageous. The relationship between behavioral unpredictability and the expected foraging success is not necessarily an indicator that determines the effect of behavioral unpredictability on expected fitness. Given the simple mechanism, state-dependent mortality may be generally relevant to the expression of behavioral unpredictability in a wide variety of systems.

## Methods

The model describes a situation in which animals forage for a fixed period and reproduce at the end of that period. It is a discrete time model dividing the prebreeding (i.e., foraging) period in *T* discrete time steps (indexed by *t*), while reproduction occurs at $t = T + 1$. Reproductive success is used as the surrogate of fitness. Foraging success is realized in each time step ($t = 1,2,\ldots ,T$), while the state *y* (i.e., surrogate of foraging success) of an individual may change accordingly at each time step. State *y* may be any variable influenced by foraging success (e.g., starvation, fat storage, size) and takes a discrete value between $y_{\text {min}}$ and $y_{\text {max}}$. For example, the state of an individual with $y=y_{\text {max}}$ will not increase even after a successful foraging event. For the analysis, $y_{\text {min}}=0$, $y_{\text {max}}=50$ and $T=50$ are used, but results are not sensitive to these values. The R script that implements the model is available in Additional file 2.

### State-dependent mortality

Two state-dependent mortality factors are considered: starvation and mass-dependent predation [22, 33]. The lower the state level, the higher the mortality due to starvation. This mortality is expressed as $e^{-\alpha y}$, $\alpha >0$ is a parameter that determines the relationship. The model also assumes that the higher the state level, the higher the predation risk. This mortality is described by a simple quadratic function ($\beta y^2$) [22]. Both $e^{-\alpha y}$ and $\beta y^2$ are the probability of death of an individual of state *y* in one time step. Combining these two factors, the probability of death for an individual of state *y* is,

2 $$\begin{aligned} d(y) = 1 - (1-e^{-\alpha y})(1-\beta y^2) \end{aligned}$$

assuming that the mortality factors act independently. In this study, the maximum mass-dependent mortality is set as 0.2 (i.e., $\beta =0.2/y_{\text {max}}^2$), and $\alpha =0.5$ (Fig. 2). These values were arbitrarily chosen, but general conclusions of the study are not altered by the specific parameter values. This study has two points: 1) the role of behavioral unpredictability over finite times in generating variation in observations and 2) the origin of nonlinearity in fitness functions. The latter point is demonstrated using linear state-dependent mortality functions because it may be misinterpreted that nonlinearity in fitness functions can result only from nonlinear state-dependent mortality (e.g., Eq. 2).

### Behavioral unpredictability

**Fig. 5:**
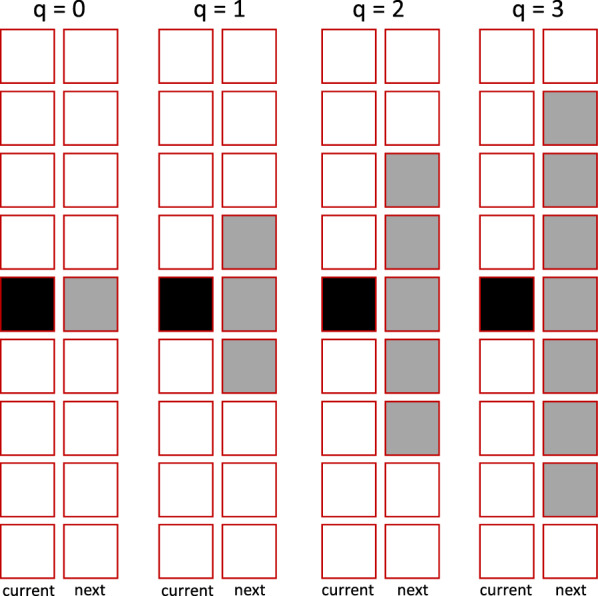
Models of behavioral unpredictability. Behaviioral unpredictability is expressed as variability in foraging outcome. The black square represents the current state, and grey squares represent possible states at the next time step for a given behavioral unpredictability, *q*. As behavioral unpredictability increases, variability in behavioral outcomes also increases

This study assumes positive correlations between behavioral unpredictability and variability in foraging outcome and weak correlations between behavioral unpredictability and expected foraging outcome (e.g., Fig. 1). That is, the model does not explicitly describe behavior, and behavioral unpredictability is expressed by the assumed correlations. Four levels of behavioral unpredictability (denoted by $q=0,1,2,3$) are considered (Fig. 5). In Fig. 5, squares represent possible states in which columns represent time, and rows indicate state *y*. The black square is the current state, and the grey squares are the possible state levels at the next time step, which varies by behavioral unpredictability, *q*. As *q* increases, variability in foraging outcome also increases (the specific model is described below). Due to environmental stochastically, there will be variability in behavioral outcome in the absence of behavioral unpredictability (Fig. 1), but this detail was not included in the model. Eliminating $q=0$ from the model does not change conclusions.

The effect of behavioral unpredictability on the expected foraging outcome is considered with the model $E(Y_{t+1})=y_t+uq$ where $Y_{t+1}$ is the random variable representing the state attained after one foraging time step when the current state is $y_t$, and *u* is the effect of behavioral unpredictability on expected behavioral outcome. Two cases are considered: $u=0$ and $u=-0.1$. The former represents the case where behavioral unpredictability has no effect on expected foraging outcome, and the latter represents the case where behavioral unpredictability negatively influence the expected behavioral outcome (e.g., Fig. 1). Behavioral unpredictability is expressed by the range of possible outcomes, $[E(Y_{t+1})-q, E(Y_{t+1})+q]$, which increases with *q* (Fig. 5). Because state *y* takes discrete values, when $E(Y_{t+1})\pm q$ are not discrete values, they are rounded.

The probability that the state level of an individual whose unpredictability level is *q* changes from *y* to *z* in one time step, $P_{q}(z|y)$, is described by a binomial distribution whose interpretation is conveniently modified. A binomial distribution is parameterized by its size parameter *N* and probability parameter *p* such that *Np* and $Np(1-p)$ are the mean and variance of the distribution, respectively. When the current state is *y* and $q=1$, there are three possible state levels at the next time step (i.e., $y-1$, *y*, and $y+1$; Fig. 5), and the probabilities associated with the three states are described by a binomial distribution with $N=2$. In particular, $P_{q=1}(y+1|y)=P_{q=1}(y-1|y)=0.25$ and $P_{q=1}(y|y)=0.5$ when the expected change is 0 (i.e. $u=0$), and this distribution is the same as a binomial distribution with $Np=1$ and $N=2$. That is, the outcome 0 for the binomial distribution is set to the lowest possible state level in the model. Similarly, $P_{q=1}(y-1|y)=0.3025$, $P_{q=1}(y|y)=0.4950$, and $P_{q=1}(y+1|y)=0.2025$ when the state level is expected to decrease by 0.1 (i.e., $u=-0.1$), which is equivalent to a binomial distribution with $Np=0.9$ and size $N=2$. For $q=2$ and $q=3$, binomial distributions with $N=4$ and $N=6$, respectively, are used. In addition, as described above, state dynamics is also constrained by $y_{\text {max}}$ and $y_{\text {min}}$.

### Optimal foraging and fitness

The function $F_q(y,t)$ describes the expected reproductive success of an individual whose state level is *y* and unpredictability level of *q* at time *t*,

3 $$\begin{aligned} F_q(y,t)=(1-d(y))\sum _{z=y_{\text {min}}}^{y_{\text {max}}} P_q(z|y)F(z,t+1)~ \end{aligned}$$

where *d*(*y*) and $P_q(z|y)$ are described above. *F*(*z*, *t*) (without a subscript) is the maximum possible expected fitness among the four levels of behavioral unpredictability such that,

4 $$\begin{aligned} F(y,t)=\text {max}\{F_0(y,t),F_1(y,t),F_2(y,t),F_3(y,t)\}~ \end{aligned}$$

and the optimal behavioral unpredictability $q^*$ emerges from Eq. 4 (e.g., $q^*=2$ when $F_2(y,t)$ is the maximum). When multiple strategies lead to the same expected fitness, the smallest *q* was chosen for convenience.

The expected fitness at $\tau$ time steps prior to reproduction, $F(y,T+1-\tau )$, is denoted as $\Phi _{\tau }(y)$ such that the terminal fitness is $\Phi _{0}(y)=F(y,T+1)$. According to the expected utility hypothesis, the curvature of $\Phi _{\tau }(y)$ will determine the effect of behavioral unpredictability at one-time step prior to $\tau$. To clearly illustrate the effect of state-dependent mortality, the terminal fitness is assumed to be linear $\Phi _{0}(y)=y/y_{\text {max}}$. Eqs. 3 and 4 reveal that *F*(*y*, *t*) may be computed when $F(y,t+1)$ is known. Therefore, the equations immediately lead to *F*(*y*, *T*) because $F(y,T+1)$ is assumed to be known. Similarly, once *F*(*y*, *T*) is known, $F(y,T-1)$ may be computed. By iterating this process, the *F*(*y*, *t*) for all possible combinations of *y* and *t* may be computed. This method is known as backward iteration [24].

### Boundary effects

Even when the expected fitness $\Phi _{\tau }(y)$ is linear, the constraints on *y* (i.e., $y_{\text {min}} \le y \le y_{\text {max}}$) will necessarily impose nonlinearity in the neighborhoods of $y_{\text {min}}$ and $y_{\text {max}}$ in $\Phi _{\tau }(y)$, that is, boundary effects. In Results, before describing the results of the full dynamic state variable model with behavioral unpredictability, isolated effects of state-dependent mortality *d*(*y*) on the curvature of $\Phi _\tau (y)$ are shown by assuming no state dynamics (i.e., only $q=0$ described in Fig. 5 is the possible outcome). Boundary effects influence $\Phi _\tau (y)$ and propagates as $\tau$ increases, even in the absence of state-dependent mortality (e.g., $d(y)=0$ for all *y*). Because the main purpose of this study is to describe the effect of state-dependent mortality, boundary effects are first eliminated, although they are necessarily included in the full model. Isolated effects of boundary effects are described in the Appendix.

As described above, the expected state change was assumed to be 0 (e.g., $E(Y_{t+1})=y_t$ when $u=0$). In addition, both positive and negative expected changes can result in nature depending on environmental conditions. For example, harsh environmental conditions [34], including density-dependent competition [35], may result in the expected net negative rewards of foraging. Results assuming positive and negative expected changes (i.e., $E(Y_{t+1})=y_t+1$ and $E(Y_{t+1})=y_t-1$) are also shown in Appendix. These changes will strengthen the boundary effects because the state of foragers will more likely constrained by the boundaries due to the expected directional change, but general conclusions are not altered by these changes.

### Supplementary information

**Additional file 1.** R script to produce Fig. 1.

**Fig. 6:**
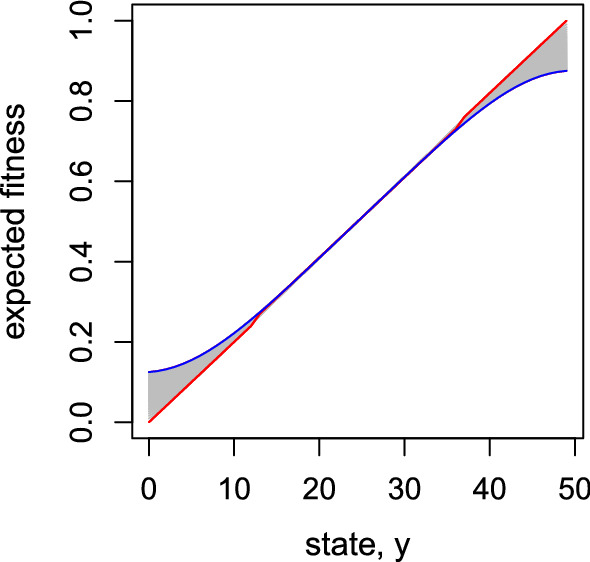
Boundary effects on expected fitness, $\Phi _{\tau }(y)$. The red line is the terminal fitness, $\Phi _{0}(y)$, and the blue line is $\Phi _{50}(y)$. Gray lines are $\Phi _{\tau }(y)$ for $1\le \tau \le 49$. $d(y)=0$ for all *y*, and $q=3$. All other details are the same as those used in Figs. 2 and 3

**Fig. 7:**
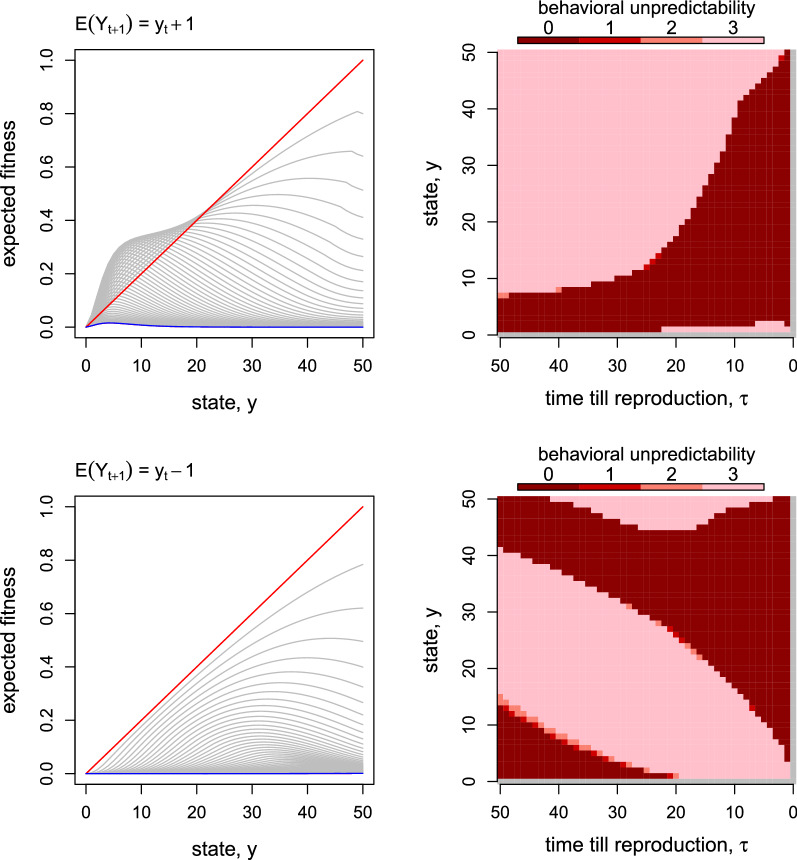
Time-dependent expected fitness (left column) and optimal behavioral unpredictability (right column). Top figures correspond with a case where the expected state change is positive ($E(Y_{t+1})=y_t+1$), and bottom figures correspond with a case where the expected state change is negative ($E(Y_{t+1})=y_t-1$). All other details are the same as those used in Fig. 5 (top row: $E(Y_{t+1})=y_t$)

**Additional file 2.** R script to produce Figs. 2, 3, 4, 6, and 7.

## Data Availability

R scripts used in the study are available in Additional files.

## Appendix

Boundary effects are briefly illustrated using the model described in the text. The model assumes no expected change in state level ($E(Y_{t+1})=y_t$), and a static level of behavioral unpredictability $q=3$ (foragers always express $q=3$). Due to the variability in the resulting state (Fig. 5), state dynamics is constrained by the boundaries. To isolate the boundary effect, state-independent mortality was assumed ($d(y)=0$ for all *y*). Under these assumptions, $\Phi _{\tau }(y)$ still becomes progressively nonlinear as $\tau$ increases (Fig. 6). In other words, the standard assumption in dynamic state variable models (i.e., state *y* is constrained by some $y_{\text {min}}$ and $y_{\text {max}}$) creates nonlinearity in the fitness function by itself.

When state *y* is expected to increase ($E(Y_{t+1})=y_t+1$) or decreases ($E(Y_{t+1})=y_t-1$), state dynamics is more strongly constrained by the boundaries, which enhances boundary effects (Fig. 7). The state-dependent mortality function used in the main analysis (Eq. 2) was also used here. When the upper boundary is more likely reached (i.e., $E(Y_{t+1})=y_t+1$), the boundary effect from the upper boundary is strengthened. That is, the region where behavioral unpredictability is selected expands from the upper bound (compare Fig. 7 top and Fig. 4 top). When the lower boundary is more likely reached ($E(Y_{t+1})=y_t-1$), the region where behavioral unpredictability is selected expands from the lower boundary (compare Fig. 7 bottom, and Fig. 4 top). However, as $\tau$ increases, the fitness function $\Phi _{\tau }(y)$ becomes largely linear (i.e., the probability of survival till reproduction is essentially 0 regardless of *y*). Under those conditions, *q* does not influence the expected fitness. As described above, when multiple strategies result in the same outcome, the smallest *q* is shown for convenience. In those cases, $q=0$ does not indicate behavioral unpredictability is selected against (e.g., the region corresponding with $q=0$ when $\tau >20$ and $y<15$ in Fig. 7 bottom).
